# Screen Time, Digital Content Quality, and Parental Mediation as Predictors of Linguistic and Pragmatic Development: Implications for Pediatric and Preventive Health

**DOI:** 10.3390/children13010157

**Published:** 2026-01-22

**Authors:** Csongor Toth, Brigitte Osser, Gyongyi Osser, Laura Ioana Bondar, Roland Fazakas, Nicoleta Anamaria Pascalau, Ramona Nicoleta Suciu, Corina Dalia Toderescu, Bombonica Gabriela Dogaru

**Affiliations:** 1 Doctoral School of Biomedical Sciences, University of Oradea, 410087 Oradea, Romania; toth.csongor1@student.uoradea.ro (C.T.); bondar.lauraioana@student.uoradea.ro (L.I.B.); gabriela.dogaru@umfcluj.ro (B.G.D.); 2 Faculty of Physical Education and Sport, “Aurel Vlaicu” University of Arad, 310130 Arad, Romania; gyongyi.osser@uav.ro; 3 Department of Biology and Life Sciences, Faculty of Medicine, “Vasile Goldiș” Western University of Arad, 310025 Arad, Romania; fazakas.roland@uvvg.ro; 4 Multidisciplinary Doctoral School, “Vasile Goldiș” Western University of Arad, 310025 Arad, Romania; 5 Department of Psycho Neuroscience and Recovery, Faculty of Medicine and Pharmacy, University of Oradea, 410087 Oradea, Romania; nicoleta.pascalau@didactic.uoradea.ro (N.A.P.); ramona_suciu@uoradea.ro (R.N.S.); 6 Faculty of Pharmacy, “Vasile Goldiș” Western University of Arad, 310045 Arad, Romania; toderescu.corina@uvvg.ro; 7 Department of Medical Rehabilitation, “Iuliu Hațieganu” University of Medicine and Pharmacy Cluj-Napoca, 410087 Cluj-Napoca, Romania; 8 Clinical Rehabilitation Hospital, 400347 Cluj-Napoca, Romania

**Keywords:** adolescent, child, communication, language development, parenting, screen time

## Abstract

**Highlights:**

**What are the main findings?**

- Higher daily screen time is consistently associated with weaker linguistic and pragmatic performance in children and adolescents, particularly in younger age groups.
- Exposure to educational digital content and more frequent parental mediation are positively associated with vocabulary, fluency, grammatical skills, and pragmatic communication.

**What are the implications of the main findings?**

- Pediatric and preventive healthcare may benefit from discussing digital media use as part of the broader developmental context of language development.
- Discussions with families may benefit from focusing not only on screen time quantity, but also digital content quality and parental involvement.

**Abstract:**

**Background/Objectives:** Although numerous studies have examined associations between screen time and early language development, less is known about how screen exposure interacts with developmental stage, digital content quality, and parental mediation across childhood and adolescence, particularly with respect to pragmatic communication. This study aimed to address these gaps by examining the joint associations of screen time, content composition, and parental mediation with multiple linguistic and pragmatic domains across a broad age range. **Methods:** A cross-sectional study was conducted with 286 Romanian participants aged 5–19 years, grouped into four developmental stages. Measures included daily screen time, proportion of educational versus recreational content, parental mediation practices, and standardized assessments of vocabulary, verbal fluency, grammatical competence, and pragmatic communication. Analyses included descriptive statistics, Pearson correlations, 4 × 3 factorial ANOVAs (age group × screen-time category), and multiple linear regression. **Results:** Higher levels of screen exposure were consistently associated with lower performance across all linguistic and pragmatic domains (r = −0.19 to −0.28, all *p* < 0.01). Participants viewing >2 h/day showed significantly weaker outcomes than those with ≤1 h/day, particularly in semantic and phonemic fluency and pragmatic communication (*p* < 0.001). Educational content correlated positively with linguistic scores, whereas recreational content showed negative associations. Parental mediation emerged as a significant positive predictor. In the regression model (R^2^ = 0.42), age (β = 0.47), parental mediation (β = 0.21), and educational content (β = 0.18) predicted better linguistic performance, while screen time (β = −0.29) predicted lower performance. **Conclusions:** The findings indicate that associations between digital media use and linguistic and pragmatic performance vary across developmental stages and contextual factors. Rather than screen time alone, digital content quality and parental mediation are associated with differences in communicative performance. These results highlight the value of a nuanced, developmentally informed perspective when considering children’s digital media environments.

## 1. Introduction

Over the past decade, digital media has become a dominant component of children’s and adolescents’ daily environments, reshaping the ways in which they learn, communicate, and interact with others. Smartphones, tablets, computers, and streaming platforms now account for a substantial proportion of leisure and educational activities, often replacing traditional forms of interaction such as reading, unstructured play, or conversational exchanges with caregivers [1,2,3,4,5]. This shift has prompted growing scientific interest in understanding how digital media influences linguistic, cognitive, and socio-emotional development. Because language acquisition relies heavily on rich, socially contingent interactions, the increasing reliance on screen-based activities raises important questions about its developmental impact [6,7,8,9,10].

Current research highlights both potential benefits and concerns associated with digital exposure. Educational digital content may support vocabulary growth, conceptual learning, and narrative skills, particularly when it incorporates clear language modeling, slower pacing, and opportunities for guided engagement [11,12,13]. Conversely, excessive or unstructured screen time has been associated with reduced conversational turn-taking, diminished attention, lower verbal fluency, and delays in expressive and receptive language development [14,15,16,17,18]. However, the literature is far from uniform. Some studies report minimal or no negative effects when digital media is used in moderation, while others suggest that the relationship depends strongly on contextual factors such as parental mediation, content quality, and the child’s developmental stage [19,20,21]. These diverging findings underscore ongoing debate about whether screen exposure itself is detrimental or whether its impact is mediated by how, when, and why children engage with digital media.

Several theoretical perspectives offer insight into these mixed results. Social interactionist frameworks emphasize the necessity of reciprocal interactions for language learning, suggesting that screen time may reduce opportunities for caregiver–child communication [12,22,23,24,25]. Cognitive load theory proposes that fast-paced or visually dense media may overwhelm children’s processing capacity, limiting linguistic integration [26,27,28]. In contrast, media learning theories argue that well-designed educational content can provide meaningful scaffolding and enhance linguistic skills, particularly for older children who possess more advanced cognitive resources [14,29,30].

Despite substantial prior research, several gaps remain. First, much of the existing literature focuses on preschool-aged children, leaving limited evidence on how digital media use relates to linguistic and pragmatic development across middle childhood and adolescence. Second, screen exposure is frequently treated as a single, undifferentiated construct, with insufficient attention to distinctions between educational and recreational content, despite theoretical reasons to expect qualitatively different effects. Third, although parental mediation is widely acknowledged as an important contextual factor, it is rarely examined simultaneously with screen time and content type across multiple linguistic domains, including verbal fluency, grammatical competence, and pragmatic communication. As a result, current evidence provides an incomplete understanding of how developmental stage, media characteristics, and family context jointly relate to language outcomes.

The present study addresses these gaps by adopting an integrative, developmentally informed approach to digital media use and language development. Specifically, it examines the associations of screen time quantity, digital content composition, and parental mediation with multiple linguistic and pragmatic domains in a broad age-stratified sample of children and adolescents aged 5 to 19 years. By extending analysis beyond early childhood and beyond global language measures, the study aims to contribute to a more nuanced understanding of how digital media use relates to communicative development across developmental stages, without assuming uniform effects across ages or contexts.

## 2. Materials and Methods

### 2.1. Study Design and Setting

This study employed a cross-sectional observational design aimed at examining the associations between screen time, digital content composition, parental mediation, and linguistic performance in children and adolescents. Data were collected at Vinga Technological High School, located in Arad County, Romania. Although formally designated as a technological high school, the institution administratively includes primary, lower secondary, and upper secondary levels, allowing the inclusion of students aged 5 to 19 years. Data collection took place between February 2024 and August 2025.

### 2.2. Participants

A total of *N* = 327 students were initially screened for participation in the study. Participants ranged in age from 5 to 19 years and were recruited from primary, lower secondary, and upper secondary classes at Vinga Technological High School (Arad County, Romania). After applying the inclusion and exclusion criteria, 286 participants were retained for the final analyses.

Participants were categorized into four developmental groups based on chronological age:

- G1: 5–8 years (*n* = 72)
- G2: 9–12 years (*n* = 72)
- G3: 13–15 years (*n* = 71)
- G4: 16–19 years (*n* = 71)

The final sex distribution was fully balanced (143 girls, 143 boys).

Participants were recruited from intact school classes. Because the class sizes at the primary, lower secondary, and upper secondary levels of Vinga Technological High School were relatively uniform, the resulting age groups (G1–G4) were naturally similar in size. No additional procedures were used to equalize the groups.

#### 2.2.1. Inclusion Criteria

Participants were eligible if they met the following criteria:

- Age between 5 and 19 years.
- Enrollment in the school during the study period.
- Typical developmental history as reported by caregivers.
- Romanian is the primary language spoken at home.
- Regular exposure to digital devices.
- Informed parental consent (for minors) or self-consent (18–19 years).

#### 2.2.2. Exclusion Criteria

Of the 327 students screened, 41 participants were excluded based on the following criteria:

- Neurodevelopmental disorders reported by parents or teachers (*n* = 9)
- Uncorrected sensory impairments (visual or hearing problems) interfering with testing (*n* = 6)
- Significant medical or neurological conditions affecting cognitive or linguistic functioning (*n* = 4)
- Non-native Romanian speakers or insufficient Romanian proficiency (*n* = 7)
- Severe behavioral or emotional difficulties preventing reliable test administration (*n* = 5)
- Incomplete data or missing responses on key variables such as screen time or linguistic tests (*n* = 10)

**Figure 1:**
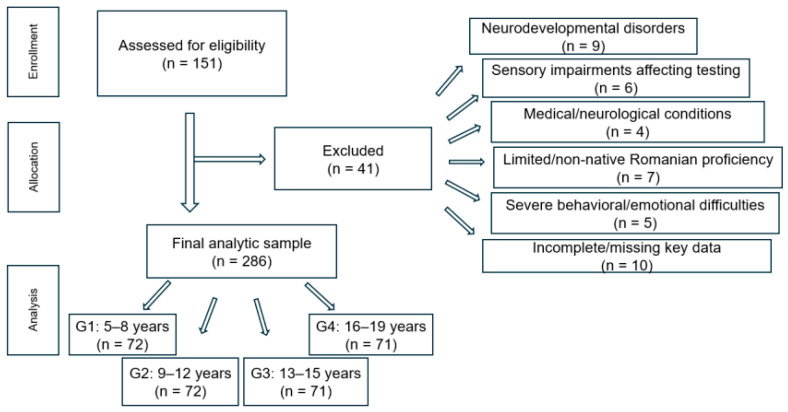
Flow diagram of participant screening, exclusions, and final analytic sample.

After these exclusions, the final analytic sample consisted of *N* = 286 participants, all with complete datasets for the variables included in this study. The exclusion process is illustrated in Figure 1.

### 2.3. Measures

All measures were selected based on their documented use in developmental and clinical research on language acquisition and communication skills, as well as their applicability to Romanian-speaking children and adolescents across a broad age range. Where standardized Romanian adaptations were available, these were used to ensure linguistic and cultural validity. Administration followed published guidelines for each instrument to maximize reliability and comparability across age groups.

#### 2.3.1. Screen Time and Digital Content Composition

Daily screen exposure was assessed using a standardized media-use questionnaire, administered as a parent-report for younger children (G1 and G2) and as a self-report for adolescents (G3 and G4), with parental verification when needed. This mixed reporting approach was selected to reflect developmental differences in self-monitoring accuracy and has been widely used in pediatric and developmental research examining digital media use. Respondents estimated the average number of minutes per day spent using digital devices, including smartphones, tablets, computers, and televisions, across typical weekdays and weekends.

To analyze content quality, participants also reported the proportion (%) of their daily screen time devoted to two categories:

- Educational digital content, such as instructional videos, school platforms, e-learning materials, documentaries, and educational applications.
- Recreational digital content, including entertainment videos, video games, social media, and streaming platforms.

These categories were defined based on media learning and cognitive load frameworks, which posit distinct linguistic and cognitive demands associated with structured instructional content versus entertainment-oriented media. Reported percentages were required to sum to 100%, ensuring mutually exclusive classification and facilitating the computation of individualized media-use profiles.

For younger children (G1–G2), screen time and content composition were reported exclusively by parents, whereas for adolescents (G3–G4), self-reports were used, with parental verification when necessary to enhance accuracy. Although parent- and self-report measures may introduce age-related measurement differences, this approach reflects standard practice in developmental research and balances feasibility, ecological validity, and developmental appropriateness. Potential age-related reporting differences were considered when interpreting cross-group comparisons.

#### 2.3.2. Parental Mediation

Parental mediation practices were measured using a four-item scale, rated on a four-point Likert format (1 = never, 4 = always). The scale was designed to capture the frequency of core mediation strategies commonly identified in the digital parenting literature and was appropriate for use across a broad developmental age range. The items assessed four key dimensions of parental mediation:

- Co-viewing and shared media engagement, reflecting joint parent–child media use;
- Rule-setting, including limits on screen time duration and content type;
- Active mediation, operationalized as discussions about digital content and its meaning;
- Monitoring and supervision of children’s online activities.

Items were averaged to yield a total parental mediation score, with higher values indicating greater parental involvement in guiding children’s digital media use. The scale focuses on behavioral frequency rather than qualitative depth, allowing consistent interpretation across age groups. Internal consistency in the present sample was acceptable, supporting its use as a continuous predictor.

Parental mediation was included as a contextual variable in the analyses to examine its role as a protective factor moderating associations between digital media exposure and linguistic and pragmatic outcomes.

#### 2.3.3. Linguistic and Pragmatic Measures

Linguistic performance was assessed using standardized and developmentally appropriate instruments selected to capture multiple complementary domains of language functioning in Romanian-speaking children and adolescents. This multidimensional approach was adopted to move beyond global language scores and to allow domain-specific analyses of vocabulary, executive-linguistic processing, grammatical competence, and pragmatic communication. The following domains were evaluated:

- Receptive vocabulary, assessed using the Romanian adaptation of the Peabody Picture Vocabulary Test (PPVT), which measures lexical comprehension through picture–word matching tasks and is widely used as an index of receptive language ability across childhood and adolescence.
- Expressive vocabulary, measured using the Romanian version of the Expressive Vocabulary Test (EVT), which evaluates lexical retrieval and expressive word knowledge and complements receptive measures by indexing productive language skills.
- Semantic verbal fluency, assessed with the Category Fluency task (e.g., animals, fruits), where participants generated as many category-related words as possible within 60 s. This task indexes semantic organization, lexical access, and executive control.
- Phonemic verbal fluency, evaluated using the Romanian adaptation of the Controlled Oral Word Association Test (COWAT), requiring participants to produce words beginning with specific letters within a fixed time. This measure primarily reflects phonological retrieval and executive functioning.
- Grammatical competence, measured with a morphosyntactic assessment battery adapted for Romanian, including sentence completion, correction, and syntactic judgment items. This battery assesses syntactic accuracy and morphological structure across development.
- Pragmatic communication, evaluated using the Pragmatic Communication Skills Scale, adapted for Romanian, which assesses the ability to use language appropriately across social and situational contexts (e.g., conversational turn-taking, contextually appropriate responses, inference-making).

For measures consisting of multiple items (receptive vocabulary, expressive vocabulary, grammatical competence, and pragmatic communication), internal consistency was evaluated using Cronbach’s α. For semantic and phonemic verbal fluency tasks, Cronbach’s α was not computed because each task yields a single performance score rather than multiple items; their inclusion was justified based on extensive evidence of test–retest and inter-rater reliability reported in the literature. All assessments were administered and scored according to standardized procedures.

#### 2.3.4. Demographic and Contextual Variables

Several demographic and contextual variables were collected to characterize the sample and control for factors known to influence linguistic development.

- Age: Recorded in years as a continuous variable and also used to categorize participants into four developmental groups (G1–G4).
- Sex: Coded as female or male, with equal representation across the final sample.
- School environment: Classified as urban or rural based on the students’ place of residence, as reported in school records.
- Parental education level: Reported separately for mothers and fathers using an ordinal scale (e.g., primary school, secondary school, high school, university). Group-level patterns reflected the dominant educational levels within each age group.
- Daily reading time: Estimated in minutes per day, representing the average amount of time the child spent reading books or school materials outside of class.
- Sleep duration: Measured as the self-reported (or parent-reported for younger children) average number of hours slept per night.

These variables were included to provide a comprehensive overview of the sample’s socio-educational context and were used descriptively to interpret group differences across developmental stages.

### 2.4. Procedure

Data collection was conducted on school premises during regular school hours. All assessments were administered individually in quiet classroom spaces or designated testing rooms to minimize distractions and ensure standardized conditions.

Before data collection, parents or legal guardians received an information sheet describing the purpose of the study, the procedures involved, and confidentiality safeguards. Written informed consent was obtained for all participants under 18 years old, and assent was collected from children capable of providing it. Participants aged 18–19 years provided their own informed consent.

The procedure followed these steps:

1. Screening and Eligibility Check: Parents completed a brief developmental and health questionnaire to confirm inclusion and exclusion criteria (e.g., language background, medical history, neurodevelopmental conditions).
2. Administration of Screen-Time and Parental Mediation Questionnaires:
  - For younger children (G1–G2), parents completed the media-use and parental mediation questionnaires.
  - For older participants (G3–G4), self-report questionnaires were administered in-class, with instructions provided by trained researchers. Parental verification was requested when necessary to ensure accuracy.
3. Linguistic Assessment: Trained examiners administered the linguistic and pragmatic measures following standardized administration procedures. Younger children were evaluated individually, using age-appropriate explanations and visual supports where relevant. Adolescents (G3–G4) completed the tests under similar standardized conditions.
4. Recording of Demographic and Contextual Variables: Age, sex, school environment (urban/rural), parental education, daily reading time, and sleep duration were recorded using school records and parental questionnaires.

The full assessment process required approximately 25–35 min per participant, depending on age and the pace at which the child completed the linguistic tasks. All procedures were conducted in accordance with institutional ethical guidelines.

### 2.5. Statistical Analysis

All statistical analyses were performed using IBM SPSS Statistics, Version 28.0 (IBM Corp., Armonk, NY, USA). Prior to analysis, the dataset was examined for missing values, outliers, and distributional properties to ensure suitability for parametric testing.

Descriptive statistics (means, standard deviations, and frequencies) were computed for all demographic, contextual, and linguistic measures. Pearson correlation coefficients were used to assess associations among screen time, digital content composition, parental mediation, and linguistic/pragmatic variables, given the continuous nature of the variables and approximately normal score distributions.

To evaluate developmental and exposure-related differences in domain-specific linguistic and pragmatic outcomes, a series of 4 × 3 factorial analyses of variance (ANOVAs) was conducted. Age group (G1–G4) and screen-time category (≤1 h/day, 1–2 h/day, >2 h/day) were included as between-subject factors. Screen-time categories were defined based on commonly cited pediatric media-use thresholds, allowing clinically meaningful group comparisons and alignment with existing literature. This analytic approach enabled examination of both main effects and Age × Screen Time interaction effects, which were theoretically expected given developmental differences in sensitivity to digital exposure.

For regression analyses, an overall linguistic performance index served as the dependent variable. This composite score was computed by standardizing and averaging the six linguistic and pragmatic measures, allowing integration of outcomes assessed on different scales. The use of a composite index reduced measurement-specific error, increased statistical power, and provided a global indicator of communicative functioning suitable for regression modeling.

A multiple linear regression analysis was conducted to examine the combined predictive contribution of age, daily screen time, proportion of educational content, and parental mediation to overall linguistic performance. All continuous predictors were mean-centered. Regression diagnostics confirmed that the assumptions of linearity, normality of residuals, homoscedasticity, independence, and absence of multicollinearity were met (all variance inflation factors < 2).

A significance level of *p* < 0.05 was applied for all statistical tests, with more stringent thresholds (e.g., *p* < 0.01) reported where appropriate. All analyses were two-tailed. Effect sizes (*η*^2^ for ANOVAs and standardized β coefficients for regression) were reported to facilitate interpretation of practical significance.

### 2.6. Ethical Considerations

The study was conducted in accordance with the principles of the Declaration of Helsinki and was approved by the Institutional Ethics Committee of Vinga Technological High School, Arad County, Romania (protocol code 1435/2, approved on 16 October 2023).

Written informed consent was obtained from all parents or legal guardians of participants under 18 years of age, and assent was collected from children capable of providing it. Adolescents aged 18–19 years provided their own informed consent prior to participation.

### 2.7. Hypotheses of the Study

Based on previous empirical findings in the domain of child and adolescent language development, digital media exposure, and family context, as well as the conceptual rationale, the present study formulated the following hypotheses. These hypotheses were also informed by descriptive and theoretical patterns commonly reported in the literature—such as the negative association between excessive screen exposure and linguistic performance, the positive role of educational digital content, and the moderating effects of parental mediation and developmental stage.

1. Screen time will be negatively associated with linguistic and pragmatic performance.
2. Exposure to educational digital content will be positively associated with linguistic outcomes, whereas recreational content will show the opposite pattern.
3. Parental mediation will positively predict linguistic and pragmatic performance.
4. Age will show a strong positive association with linguistic performance, reflecting developmental progression across groups.
5. The negative association between screen time and linguistic performance will be stronger in younger children than in adolescents.
6. When considered simultaneously, age, screen time, parental mediation, and digital content type will jointly predict overall linguistic performance.

## 3. Results

### 3.1. Descriptive Characteristics of the Sample

The final sample consisted of *N* = 286 participants aged 5 to 19 years, distributed across four developmental groups: G1 (5–8 years), G2 (9–12 years), G3 (13–15 years), and G4 (16–19 years). The sex distribution was balanced (143 girls, 143 boys), ensuring comparability across groups. The numerical distribution by age group and sex is presented in Table 1.

The descriptive analysis of demographic and contextual variables showed moderate variation between age groups in terms of school environment, daily reading time, and sleep duration. Detailed values for each group are presented in Table 2.

The main psychological variables included receptive and expressive vocabulary, semantic and phonemic verbal fluency, grammatical score, and pragmatic communication. All measures displayed adequate variability and wide score ranges, confirming their suitability for subsequent analyses. Screen time and digital content composition also showed substantial between-participant variability. Descriptive statistics for all variables are presented in Table 3.

To further characterize patterns of digital media use, participants were categorized into three screen-time groups (≤1 h/day, 1–2 h/day, >2 h/day). As shown in Table 4, screen time increased progressively with age. The highest proportions of exposure exceeding 2 h/day were observed in G3 and G4.

### 3.2. Correlations Among Study Variables

Pearson correlation coefficients among the main variables are presented in Table 5. The strongest negative associations were observed between screen time and parental mediation (r = −0.35, *p* < 0.01) and between screen time and pragmatic communication (r = −0.28, *p* < 0.01). Screen time also showed consistent small-to-moderate negative correlations with all linguistic variables (r values between −0.19 and −0.27, all *p* < 0.01).

In contrast, the linguistic measures were strongly intercorrelated. The highest positive correlations were found between receptive and expressive vocabulary (r = 0.62, *p* < 0.01), receptive vocabulary and semantic fluency (r = 0.58, *p* < 0.01), and grammatical score and pragmatic communication (r = 0.54, *p* < 0.01), confirming the expected convergence of verbal competencies.

Educational content correlated positively with linguistic variables (r = 0.15–0.21, *p* < 0.05/0.01), whereas recreational content exhibited the opposite pattern, showing small negative associations with the same indicators (r = −0.16 to −0.23, *p* < 0.05/0.01). As expected, educational and recreational content were strongly inversely related (r = −0.58, *p* < 0.01).

Overall, the correlation structure reflects three consistent patterns:

- strong interrelations among linguistic measures,
- complementary associations of educational versus recreational content,
- a systematic negative association between screen time and linguistic/pragmatic performance.

These associations provide a robust foundation for the factorial and predictive analyses presented in subsequent sections.

### 3.3. Analysis of Group Differences

To examine differences in linguistic and pragmatic performance as a function of developmental stage and level of digital exposure, a series of 4 × 3 factorial ANOVAs was conducted. Age group (G1–G4) and screen-time category (≤1 h/day, 1–2 h/day, >2 h/day) were treated as between-subject factors. Dependent variables included receptive and expressive vocabulary, semantic and phonemic fluency, grammatical score, and pragmatic communication.

#### 3.3.1. Main Effects and Interactions

The analyses revealed significant main effects of screen time on all linguistic and pragmatic variables (Table 6). Participants exposed to more than 2 h of screen time per day consistently obtained lower scores than those in the ≤1 h/day and 1–2 h/day categories. The largest differences emerged for semantic and phonemic fluency and for pragmatic communication, whereas receptive and expressive vocabulary showed smaller but still significant differences across exposure levels.

Significant main effects of age were also observed for all variables, reflecting a clear developmental progression from G1 to G4. Group G1 showed the lowest overall performance, G2 demonstrated stable increases, G3 exhibited marked improvements, particularly in fluency-related measures, and G4 reached the highest levels, with smaller incremental gains relative to G3, suggesting stabilization during adolescence. Age was the strongest predictor across all outcomes, consistent with normative developmental and educational progression. Accordingly, associations between screen exposure and language outcomes should be interpreted as age-contingent rather than age-independent.

Significant Screen Time × Age interactions were found for most outcome variables, indicating that the impact of screen time varied across developmental stages. The differences between screen-time categories were more pronounced in the younger groups (G1 and G2), whereas in adolescents (G3 and G4), the negative effects were attenuated but remained detectable.

#### 3.3.2. Interaction Effects (Screen Time × Age)

Significant Screen Time × Age interactions were observed for most linguistic and pragmatic measures, indicating that the association between digital exposure and performance differed across developmental stages. Across variables, the largest contrasts between screen-time categories (≤1 h/day vs. >2 h/day) were observed in the younger age groups (G1 and G2). In older participants (G3 and G4), the negative associations related to higher exposure remained evident but were comparatively smaller in magnitude.

Table 7 presents illustrative group means for three outcomes that showed particularly strong interaction effects—semantic fluency, phonemic fluency, and pragmatic communication. Across all variables, performance declined systematically with increasing daily screen time, with steeper reductions observed in the younger age groups. In older participants, higher screen exposure was also associated with lower scores, although group differences were less pronounced.

Supplementary File S1 illustrates the interaction between age group and screen-time exposure using a standardized composite linguistic performance score. Across all three exposure categories, linguistic performance increased progressively from G1 to G4, reflecting the expected developmental trajectory. However, participants in the >2 h/day category consistently showed lower performance than those with ≤1 h/day or 1–2 h/day of daily screen use. The separation between the performance curves was greatest in the younger age groups (G1 and G2), indicating heightened sensitivity to excessive screen exposure during early developmental stages, whereas in G3 and G4, the differences between exposure categories became smaller, although still evident.

Supplementary File S2 illustrates group differences across linguistic and pragmatic variables as a function of screen-time category. For each measure, scores decreased progressively from the ≤1 h/day group to the >2 h/day group, indicating a consistent negative association between higher levels of digital exposure and language performance. The decline was most pronounced for semantic and phonemic fluency, grammatical performance, and pragmatic communication, whereas receptive and expressive vocabulary showed smaller but still consistent reductions. These graphical patterns reinforce the ANOVA findings, highlighting a systematic disadvantage associated with daily screen use exceeding 2 h.

### 3.4. Post Hoc Analyses

Tukey HSD post hoc comparisons revealed significant pairwise differences between screen-time categories for most linguistic and pragmatic variables. The largest and most consistent contrasts occurred between the ≤1 h/day and >2 h/day groups, with the >2 h/day category showing significantly lower performance across all domains. Effect sizes for these contrasts were small to moderate (Cohen’s d = 0.32–0.58), indicating meaningful practical differences. All *p*-values reported are Tukey-adjusted, ensuring appropriate control for multiple comparisons.

Significant differences between the 1–2 h/day and >2 h/day groups were observed for semantic fluency, phonemic fluency, and pragmatic communication (d = 0.26–0.44), suggesting that these abilities are particularly sensitive to increases in daily digital exposure. In contrast, receptive and expressive vocabulary showed only modest differences and were significant only for the ≤1 h/day vs. >2 h/day comparison (d ≈ 0.30).

For grammatical performance, all three screen-time categories differed from one another in a linear fashion (≤1 h/day > 1–2 h/day > 2 h/day), with small-to-moderate effect sizes (d = 0.24–0.49), supporting a graded association between greater exposure and lower performance.

Regarding age, significant developmental gains were observed between G1 and G2 and between G2 and G3 (d = 0.45–0.70), reflecting rapid developmental progression. Differences between G3 and G4 were smaller (d = 0.18–0.32), consistent with the gradual stabilization of linguistic performance during later adolescence. Table 8 summarizes all pairwise contrasts.

Effect sizes for significant contrasts were generally small to moderate, indicating that, while the effects of screen time are statistically reliable, they also bear practical relevance for children’s linguistic and pragmatic development.

### 3.5. Multiple Regression Model

A multiple regression analysis was conducted to examine the joint contribution of demographic and digital media–related factors to overall linguistic performance. Screen time, age, educational content, and parental mediation were entered simultaneously as independent variables. The final model explained a significant proportion of the variance in the composite linguistic score (R^2^ = 0.42, adjusted R^2^ = 0.41, *p* < 0.001), indicating that the predictors collectively accounted for substantial individual differences in linguistic outcomes.

Age showed the strongest positive association with linguistic performance (β = 0.47, *p* < 0.001), consistent with expected developmental differences across childhood and adolescence. Parental mediation (β = 0.21, *p* = 0.001) and the proportion of educational digital content (β = 0.18, *p* = 0.003) also contributed positively, underscoring the supportive role of structured digital guidance and exposure to higher-quality content.

In contrast, screen time was a robust negative association (β = −0.29, *p* < 0.001), indicating that higher daily digital exposure was associated with lower linguistic performance, even after controlling for age and contextual factors.

Model diagnostics confirmed that all regression assumptions were met. Variance Inflation Factor (VIF) values were low (all < 2), indicating the absence of multicollinearity. Standardized residuals revealed no evidence of heteroscedasticity or deviation from normality, and Cook’s distance values were within acceptable limits, suggesting no influential outliers. Predictors were mean-centered to facilitate interpretation and reduce redundancy.

Confidence intervals confirmed the stability and precision of the estimated effects. Age showed a robust positive association with linguistic performance (95% CI ≈ 0.39–0.54), while screen time demonstrated a consistent negative effect (95% CI ≈ −0.36 to −0.21). Parental mediation (95% CI ≈ 0.09–0.32) and educational content (95% CI ≈ 0.06–0.29) also presented positive and statistically reliable confidence intervals, reinforcing their consistent association with linguistic outcomes within the model. Table 9 presents the regression coefficients.

To address the multidimensional nature of linguistic abilities, additional multiple regression analyses were conducted separately for each linguistic domain, using the same set of predictors (age, screen time, educational content, and parental mediation). As shown in Table 10, the pattern of associations was largely consistent across receptive vocabulary, expressive vocabulary, verbal fluency, grammatical competence, and pragmatic communication.

Accordingly, the composite linguistic score is interpreted as a global indicator of communicative functioning that captures shared variance across domains, while domain-specific analyses confirm that the observed associations are not driven by an assumption of strict unidimensionality or by age-related variance alone.

### 3.6. Internal Consistency of Measures (Reliability)

Internal consistency was evaluated only for linguistic measures that consisted of multiple items and therefore met the assumptions required for computing Cronbach’s α. Receptive and expressive vocabulary showed acceptable to good internal consistency (α = 0.78–0.84). Grammatical competence demonstrated adequate reliability (α = 0.81), and pragmatic communication yielded excellent internal consistency (α = 0.89), indicating strong construct stability.

For semantic and phonemic verbal fluency, Cronbach’s α was not computed because each fluency task yields a single total score rather than multiple items. As such, internal consistency indices are not applicable. These tasks have well-established reliability in the scientific literature and were administered using standardized procedures.

Overall, the reliability indices support the psychometric robustness of the multi-item linguistic measures used in this study (Table 11).

## 4. Discussion

The present study examined how screen time, digital content composition, parental mediation, and developmental stage jointly contribute to linguistic and pragmatic performance in children and adolescents. The findings largely supported all hypotheses and aligned with the broader literature on digital media use and language development.

A central challenge in interpreting these findings is disentangling media-related associations from normative developmental changes across childhood and adolescence. Language abilities, educational exposure, and media autonomy increase markedly with age, and age was the dominant predictor of all outcomes in the present study. Although screen exposure variables were associated with several language measures after statistical adjustment for age, these associations should not be interpreted as developmentally independent effects but rather as patterns embedded within broader age-related developmental and educational contexts.

### 4.1. Screen Time and Linguistic Performance

The present findings align with a broad body of literature indicating that higher levels of daily screen exposure are associated with weaker linguistic and pragmatic abilities across childhood and adolescence. Previous research has consistently linked increased screen use to reduced vocabulary, verbal fluency, grammatical proficiency, and pragmatic competence, often interpreted as reflecting the displacement of language-rich experiences such as caregiver–child conversations, shared reading, and interactive play [14,16,31].

From a cognitive perspective, linguistic domains that rely on rapid lexical access and executive control—particularly semantic and phonemic fluency—may be especially sensitive to excessive digital exposure. Prior studies suggest that high screen engagement may place demands on attentional regulation, working memory, and cognitive flexibility, processes that are central to efficient word retrieval and verbal organization [9,32,33,34,35]. In addition, pragmatic communication appears vulnerable to reduced opportunities for socially contingent interaction, a mechanism emphasized by social interactionist accounts of language development [15,36,37].

### 4.2. The Role of Educational vs. Recreational Digital Content

A growing body of research suggests that the quality and purpose of digital media exposure may be as important as, or more important than, overall screen time quantity when considering language-related outcomes. In this context, educational digital content has been associated with comparatively stronger linguistic and pragmatic profiles, whereas recreational or entertainment-focused content tends to show less favorable associations [11,12,13,38].

Educational media typically incorporates structured linguistic input, coherent narrative organization, and opportunities for guided engagement—features that have been linked to vocabulary growth, narrative competence, and higher-order language skills, particularly when content is characterized by slower pacing, repetition, and explicit language modeling [22,30,39,40]. Such characteristics may support linguistic processing by reducing cognitive load and facilitating the integration of verbal information.

By contrast, recreational digital content is often fast-paced and visually dense, placing greater demands on attentional resources and limiting opportunities for sustained verbal engagement. Prior research has associated these features with increased cognitive load, reduced parent–child discussion, and fewer opportunities for reflective processing during media use [2,21,24,36,41,42].

Taken together, these findings highlight the importance of distinguishing between content types when examining associations between digital media and language development. Rather than viewing screen exposure as uniformly detrimental, the evidence supports a more nuanced perspective in which educational content is linked to comparatively stronger linguistic and pragmatic outcomes when considered alongside exposure level and contextual factors.

### 4.3. Parental Mediation as a Contextual Factor

Parental mediation represents a key contextual dimension of children’s digital media environments and has been consistently associated with more favorable linguistic and pragmatic outcomes. Prior research indicates that higher levels of parental involvement during media use are linked to stronger vocabulary, fluency, grammatical abilities, and communicative skills, as well as to lower overall screen exposure [19,29,43,44,45].

Mediation strategies such as co-viewing, discussing digital content, setting boundaries, and guiding children’s interpretations have been described as contexts in which children are more likely to engage actively with linguistic information during screen-based activities. These practices have been associated with increased conversational engagement and richer opportunities for verbal interaction, supporting vocabulary growth and language comprehension [9,29,38,46,47,48,49].

Within the present study, parental mediation remained associated with linguistic performance even when considered alongside age, screen time, and content composition, suggesting that it constitutes an independent contextual correlate of language-related outcomes. This pattern aligns with broader research emphasizing the role of responsive parental communication in shaping enriched linguistic environments across both digital and non-digital contexts [47,50,51].

Overall, these results suggest that parental mediation is an important contextual factor associated with how children engage with digital media. Rather than indicating a protective effect, the findings highlight parental mediation as a dimension of the media environment that co-occurs with differences in linguistic and pragmatic performance.

### 4.4. Developmental Differences and Age Effects

Developmental progression emerged as a central dimension underlying linguistic and pragmatic performance across the sample, reflecting the well-established trajectory of language maturation from early childhood through adolescence. Extensive research has documented age-related refinement in vocabulary, verbal fluency, grammatical competence, and pragmatic skills as children advance through successive educational and developmental stages [52,53,54,55].

Consistent with this literature, the most pronounced developmental gains were observed from early childhood into middle childhood and early adolescence, a period characterized by rapid growth in both lexical knowledge and executive–linguistic processes [56,57,58,59]. By contrast, later adolescence appeared to reflect a phase of relative stabilization, in which further improvements were more incremental, aligning with evidence that foundational linguistic skills are largely consolidated by early adolescence [54,58].

The developmental stage also shaped the manner in which digital media exposure related to linguistic outcomes. Younger children demonstrated greater sensitivity to higher levels of screen exposure, whereas adolescents showed comparatively attenuated differences across exposure levels. Similar age-dependent patterns have been reported in previous studies, suggesting that early childhood may represent a particularly vulnerable period for the displacement of conversational, interactive, and language-rich experiences [9,24,36,60].

As children mature, increasing cognitive control, autonomy, and diversification of language-learning contexts may partially buffer against the effects of excessive digital exposure [12,61]. Overall, these findings underscore the importance of considering the developmental stage when evaluating the influence of digital media on linguistic outcomes. The results suggest that interventions and guidelines may need to be tailored more specifically to early childhood, where the potential for both risk and benefit appears most pronounced.

### 4.5. Developmental Sensitivity to Screen Exposure

Accumulating evidence suggests that the relationship between screen exposure and linguistic development varies as a function of developmental timing. Early childhood, in particular, appears to represent a period of heightened sensitivity during which environmental inputs—including digital media—may exert stronger associations with language-related outcomes [14,36,62,63].

Several developmental mechanisms may account for this pattern. During early childhood, language acquisition depends heavily on face-to-face interaction, joint attention, and caregiver scaffolding, which provide rich opportunities for conversational turn-taking, feedback, and socially contingent input [64,65,66,67]. Excessive screen exposure may displace these foundational learning contexts, limiting children’s access to the interactive experiences most critical for early language growth. Consistent with this view, younger children have been shown to benefit less from non-interactive or fast-paced digital media and may experience difficulty transferring on-screen information to real-world communicative contexts [6,16,68,69].

By contrast, adolescents may demonstrate greater resilience to the linguistic correlates of screen exposure. Developmental advances in metacognition, cognitive flexibility, and selective attention allow older youth to engage more strategically with digital content [70,71,72]. In addition, adolescents typically draw on a broader range of language-learning contexts—including peer interactions, academic discourse, and extracurricular activities—which may buffer against the displacement effects observed in younger children [2,9,73,74].

Together, these results underscore the importance of considering developmental timing when evaluating the effects of digital media. They suggest that early childhood may represent a period of heightened sensitivity to digital media exposure, warranting particular attention in future guideline development.

### 4.6. Combined Multivariate Model of Linguistic Skills

The multivariate findings underscore that linguistic performance is associated with a constellation of developmental, behavioral, and contextual factors rather than any single influence in isolation. Age, screen time, educational content, and parental mediation jointly accounted for a substantial proportion of variance in overall linguistic performance, reflecting a multidimensional pattern consistent with contemporary developmental frameworks that emphasize the interplay of cognitive maturation, environmental input, and media ecology [75,76,77].

Within this model, age emerged as the strongest correlate, mirroring well-established developmental progression across linguistic domains. At the same time, higher levels of daily screen exposure remained negatively associated with linguistic performance after accounting for age and content composition, consistent with prior multivariate research demonstrating associations between screen time and language outcomes beyond demographic and contextual factors [6,15,78,79].

Educational digital content and parental mediation were positively associated with linguistic performance, indicating that structured media exposure and adult co-engagement co-occur with comparatively stronger language-related outcomes. This pattern aligns with previous research linking educational media and parental involvement to enriched linguistic environments during digital media use, without implying causal effects [80,81].

The presence of both positive and negative correlates within the model underscores the complexity of associations between digital media use and language development. Rather than viewing screen time as uniformly harmful or educational media as uniformly beneficial, the findings support a nuanced, ecologically informed perspective in which language-related outcomes are associated with combinations of exposure level, content characteristics, and family context.

Overall, the multivariate model reinforces the view that language development is a multifactorial process associated with developmental stage, digital media habits, and the broader family environment. These results may help inform future longitudinal and experimental research aimed at clarifying causal mechanisms and developmental trajectories in contemporary media contexts.

### 4.7. Implications for Clinical Practice

Although the present findings reveal consistent associations between digital media use, parental mediation, and linguistic and pragmatic performance, they should be interpreted cautiously in clinical contexts. Given the cross-sectional design, absence of clinical thresholds, and small-to-moderate effect sizes, the results do not support diagnostic screening, causal inference, or prescriptive intervention strategies.

Instead, the findings may be most relevant for contextualizing developmental assessments in pediatric, speech–language, and educational settings. Information about children’s digital media habits—including approximate screen time, dominant content types, and the degree of parental involvement—may provide useful background when clinicians are evaluating language or communication concerns, particularly in younger children.

Importantly, the observed associations should be understood as descriptive correlates rather than indicators of impairment or risk. Elevated screen exposure, lower parental mediation, or higher recreational content use should not be interpreted as clinical markers but rather as environmental characteristics that may co-occur with variation in linguistic performance.

The findings also underscore the value of developmentally sensitive conversations with families about media use as part of a broader discussion of children’s learning environments. Emphasis may be placed on balancing digital activities with language-rich interactions—such as shared reading, conversation, and play—without implying that specific screen-time limits or content choices constitute evidence-based clinical recommendations.

Overall, the present results support a cautious, contextual, and non-prescriptive role for digital media considerations in clinical practice, while highlighting the need for longitudinal and experimental research before clinical screening tools or preventive guidelines can be justified.

### 4.8. Research Limitations

Although this study provides valuable insights into the relationships between digital media use and linguistic development, several limitations should be acknowledged when interpreting the findings. First, the cross-sectional design restricts the ability to draw causal conclusions. While the results demonstrate clear associations between screen exposure, content type, parental mediation, and linguistic skills, the directionality of these relationships cannot be definitively established. It remains possible that children with weaker linguistic abilities are more inclined to use screens, or that families with higher screen exposure differ in other unmeasured ways. Longitudinal or experimental studies are needed to clarify causal pathways. Moreover, because age captures multiple overlapping developmental processes (e.g., linguistic maturation, schooling, and increasing autonomy), age-related developmental change cannot be fully disentangled from media-related associations in this cross-sectional design.

Second, measures of screen time and digital content composition relied on parent report in younger children and self-report in adolescents, without independent validation against objective usage data. Although this mixed-report approach is common in developmental research and developmentally appropriate, it may introduce age-related measurement bias due to differences in parental awareness, recall accuracy, and adolescents’ self-monitoring abilities. Consequently, comparisons across age groups should be interpreted with caution, particularly with respect to absolute levels of screen exposure. Moreover, subjective reports may be influenced by recall errors or social desirability effects, potentially leading to under- or overestimation of actual media use.

Third, digital content quality was operationalized using a dichotomous classification into educational versus recreational content. While this distinction is theoretically grounded and informative, it necessarily oversimplifies the heterogeneity of digital media experiences. Educational and recreational media vary widely in pacing, interactivity, narrative structure, and linguistic richness—dimensions that were not captured in the present study. In addition, requiring participants to allocate 100% of screen time across these two categories imposes a compositional constraint that may artificially inflate inverse correlations between content types. As such, associations involving content composition should be interpreted as indicators of relative exposure patterns rather than precise estimates of independent effects.

Fourth, while the sample size was robust and covered a broad age range, the study did not explicitly control for several contextual variables that may influence linguistic development, such as socioeconomic status, home literacy environment, or cognitive ability. These factors may interact with digital media use in complex ways and could help refine predictive models in future research.

Fifth, although the linguistic measures used were psychometrically reliable and covered multiple domains, they assessed structured aspects of language functioning and may not fully capture broader communicative competencies, such as discourse-level skills, conversational reciprocity, or socio-emotional communication. More ecologically valid assessments—such as naturalistic language samples—may provide a more comprehensive understanding of real-world communication.

In addition, although a composite linguistic score was used for parsimony in multivariate modeling, domain-specific regression analyses demonstrated that the observed associations were consistent across linguistic domains and were not driven by an assumption of strict unidimensionality or by age-related variance alone.

Finally, the study was conducted within a single cultural and educational context, which may limit generalizability. Media practices, parenting norms, and language-learning environments vary across cultures, and the impact of digital exposure may differ accordingly.

Despite these limitations, the findings contribute meaningfully to the growing body of literature on digital media and language development and highlight directions for more refined future research.

### 4.9. Future Research Directions

The findings of this study open several avenues for future research aimed at deepening our understanding of how digital media shapes linguistic and pragmatic development across childhood and adolescence. First, longitudinal studies are needed to establish causal relationships and developmental trajectories. Following children over time would help clarify whether early screen exposure has lasting effects on language outcomes or whether these effects diminish as children mature and gain compensatory skills.

Second, future work should incorporate more fine-grained and objective assessments of digital media use. While this study relied on parent and self-reported screen time, emerging technologies—such as real-time usage tracking, app activity logs, and passive sensing—can provide more precise and ecologically valid measures of screen behavior, while reducing age-related reporting bias.

Third, additional research should explore the mechanisms through which educational and recreational media exert their effects. Experimental designs comparing specific content features (e.g., narrative complexity, dialogic structure, linguistic density, visual speed) would help identify which characteristics support or hinder language learning and guide the design of more effective educational media.

Fourth, future studies should examine the role of family dynamics and broader environmental factors in moderating the relationship between media use and language development. Variables such as socioeconomic status, home literacy environment, parenting styles, and family communication patterns may interact with screen exposure in complex ways and warrant integration into multivariate models.

Fifth, cross-cultural research is needed to determine whether the patterns observed in the present study generalize across diverse cultural and linguistic contexts. Comparative research could illuminate universal mechanisms versus culture-specific influences.

Finally, future studies may benefit from expanding the scope of linguistic assessment. Incorporating naturalistic language samples, conversational analysis, and discourse-level evaluations would provide richer insights into how digital media influences the complexities of real-world communication. Given that pragmatic communication showed notable sensitivity to screen exposure in this study, such ecologically grounded measures may be particularly informative.

Taken together, these directions highlight the need for interdisciplinary, methodologically diverse, and culturally inclusive research to more fully understand the evolving relationship between digital media and linguistic development.

## 5. Conclusions

This study contributes to the literature on digital media and language development by examining how screen time, digital content quality, parental mediation, and developmental stage are jointly associated with linguistic and pragmatic performance across childhood and adolescence. Rather than suggesting uniform effects of screen exposure, the findings highlight the importance of considering developmental timing and contextual factors when evaluating associations between digital media use and communicative outcomes.

By extending analysis beyond early childhood and beyond global language measures, the study underscores that digital media use relates differently to language development depending on age, content composition, and family involvement. These results support a more nuanced, integrative perspective on children’s media environments, in which screen time quantity alone provides an incomplete account of language-related outcomes.

Importantly, the cross-sectional design precludes causal inference, and the observed associations should be interpreted as descriptive rather than explanatory, potentially reflecting bidirectional relationships. Future research using longitudinal and experimental approaches, objective assessments of media use, and more fine-grained measures of communicative behavior will be essential for clarifying causal mechanisms and developmental trajectories. Such work may further refine theoretical models of media use and inform evidence-based guidance that is sensitive to developmental stage and contextual variability.

## Figures and Tables

**Table 1:** Distribution of the Sample by Age Group and Sex (*N* = 286).

Age Group	Age Interval (Years)	*N* (Total)	% of Sample	Girls (*n*)	Boys (*n*)
G1	5–8	72	25.2%	36	36
G2	9–12	72	25.2%	36	36
G3	13–15	71	24.8%	36	35
G4	16–19	71	24.8%	35	36
Total	5–19	286	100%	143	143

Note: Sex distribution was balanced across all groups.

**Table 2:** Demographic and Contextual Characteristics of the Sample (*N* = 286).

Indicator	Total	G1 (5–8)	G2 (9–12)	G3 (13–15)	G4 (16–19)
Age, M (SD)	12.1 (4.6)	6.7 (1.0)	10.3 (1.2)	14.1 (0.8)	17.3 (0.9)
School environment (urban/rural), *n* (%)	210/76 (73/27)	52/20	53/19	52/19	53/18
Parental education (mother) ^1^	Mostly secondary–university	High school	High school	High school	High school
Parental education (father) ^1^	Mostly secondary–university	High school	High school	High school	High school
Daily reading (min/day), M (SD)	21.4 (17.2)	25.6 (18.9)	22.5 (17.1)	19.3 (15.6)	17.8 (14.9)
Sleep duration (hours/night), M (SD)	8.3 (1.2)	9.4 (0.7)	8.9 (0.9)	7.9 (1.0)	7.4 (1.1)

^1^ Parental education was measured on an ordinal scale. In the total sample, most parents reported education levels ranging from secondary school to university. Group values reflect the dominant category in each subgroup.

**Table 3:** Descriptive Statistics of the Main Variables.

Variable	Total M (SD)	G1	G2	G3	G4	Min–Max
Screen time (min/day)	156 (62)	102 (45)	138 (51)	176 (58)	200 (63)	15–380
Educational content (%)	32 (18)	41 (20)	36 (17)	28 (16)	23 (14)	0–100
Recreational content (%)	68 (18)	59 (20)	64 (17)	72 (16)	77 (14)	0–100
Parental mediation	2.4 (0.9)	2.9 (0.8)	2.6 (0.8)	2.2 (0.9)	1.8 (0.8)	1–4
Receptive vocabulary	98.6 (14.5)	97.8 (13.2)	100.4 (14.1)	99.2 (14.9)	97.1 (15.3)	65–131
Expressive vocabulary	42.5 (9.1)	41.2 (8.5)	43.7 (8.9)	42.8 (9.4)	41.9 (9.8)	20–67
Semantic fluency	17.9 (6.2)	12.3 (4.3)	17.8 (5.1)	20.4 (5.5)	21.6 (5.7)	4–34
Phonemic fluency	14.7 (5.8)	9.4 (3.6)	13.9 (4.8)	16.5 (5.7)	18.3 (6.1)	3–33
Grammatical score	7.8 (2.2)	6.2 (1.8)	7.6 (2.0)	8.4 (2.1)	9.0 (2.3)	2–13
Pragmatic communication	31.4 (7.9)	28.9 (7.2)	30.8 (7.3)	32.7 (7.5)	33.8 (7.6)	16–48

**Table 4:** Distribution of Screen Time Categories by Age Group.

Group	≤1 h/day	1–2 h/day	>2 h/day	Total
G1 (5–8)	28 (38.9%)	25 (34.7%)	19 (26.4%)	72
G2 (9–12)	20 (27.8%)	27 (37.5%)	25 (34.7%)	72
G3 (13–15)	15 (21.1%)	23 (32.4%)	33 (46.5%)	71
G4 (16–19)	12 (16.9%)	22 (31.0%)	37 (52.1%)	71
Total	75 (26.2%)	97 (33.9%)	114 (39.9%)	286

**Table 5:** Correlation Matrix of Linguistic, Screen-Time, and Contextual Measures (*N* = 286).

Variable	1	2	3	4	5	6	7	8	9	10
1. Screen time (min/day)	—									
2. Educational content (%)	−0.32 **	—								
3. Recreational content (%)	0.41 **	−0.58 **	—							
4. Parental mediation	−0.35 **	0.29 **	−0.34 **	—						
5. Receptive vocabulary	−0.27 **	0.18 *	−0.23 **	0.31 **	—					
6. Expressive vocabulary	−0.22 **	0.16 *	−0.20 **	0.28 **	0.62 **	—				
7. Semantic fluency	−0.25 **	0.21 **	−0.19 **	0.26 **	0.58 **	0.55 **	—			
8. Phonemic fluency	−0.19 **	0.15 *	−0.16 *	0.22 **	0.47 **	0.44 **	0.53 **	—		
9. Grammatical score	−0.24 **	0.18 *	−0.20 **	0.25 **	0.55 **	0.52 **	0.49 **	0.46 **	—	
10. Pragmatic communication	−0.28 **	0.19 **	−0.23 **	0.29 **	0.61 **	0.58 **	0.52 **	0.49 **	0.54 **	—

Note: * *p* < 0.05; ** *p* < 0.01.

**Table 6:** Factorial ANOVA (4 × 3)—Main Effects and Interactions.

Variable	Screen Time Effect (*F*, *p*, *η*^2^)	Age Effect (*F*, *p*, *η*^2^)	Interaction (*F*, *p*, *η*^2^)
Receptive vocabulary	8.72, *p* < 0.001, 0.07	12.45, *p* < 0.001, 0.10	3.15, *p* = 0.012, 0.03
Expressive vocabulary	6.38, *p* = 0.002, 0.05	10.11, *p* < 0.001, 0.08	2.89, *p* = 0.023, 0.03
Semantic fluency	15.62, *p* < 0.001, 0.12	18.93, *p* < 0.001, 0.14	4.77, *p* < 0.001, 0.05
Phonemic fluency	13.55, *p* < 0.001, 0.11	17.28, *p* < 0.001, 0.13	4.22, *p* < 0.001, 0.05
Grammatical score	9.82, *p* < 0.001, 0.08	20.14, *p* < 0.001, 0.15	3.45, *p* = 0.008, 0.03
Pragmatic communication	14.23, *p* < 0.001, 0.11	16.01, *p* < 0.001, 0.13	5.01, *p* < 0.001, 0.06

Note: *η*^2^ = effect size (0.01 = small, 0.06 = medium, 0.14 = large). *F* = F-statistic, *p* = probability value.

**Table 7:** Group Means for Interaction Effects (Semantic and Phonemic Fluency, Pragmatic Communication).

Variable	G1 ≤1 h	G1 1–2 h	G1 >2 h	G4 ≤1 h	G4 1–2 h	G4 >2 h	Observation
Semantic fluency	13.4	12.9	10.5	22.1	21.3	19.8	Clear pattern
Phonemic fluency	10.2	9.7	8.1	19.0	18.4	17.0	Gradual decline
Pragmatic communication	30.1	28.4	25.7	35.4	34.1	32.2	Pronounced differences

**Table 8:** Summary of Post Hoc Comparisons (Tukey HSD).

Variable	≤1 h vs. 1–2 h	≤1 h vs. >2 h	1–2 h vs. >2 h	G1 vs. G2	G2 vs. G3	G3 vs. G4
Receptive vocabulary	n.s.	*p* < 0.01	n.s.	*p* < 0.001	*p* < 0.01	n.s.
		(d = 0.30)		(d = 0.62)	(d = 0.40)	
Expressive vocabulary	n.s.	*p* < 0.01	n.s.	*p* < 0.001	*p* < 0.01	n.s.
		(d = 0.28)		(d = 0.55)	(d = 0.38)	
Semantic fluency	*p* < 0.05	*p* < 0.001	*p* < 0.01	*p* < 0.001	*p* < 0.01	*p* < 0.05
	(d = 0.22)	(d = 0.58)	(d = 0.35)	(d = 0.70)	(d = 0.42)	(d = 0.25)
Phonemic fluency	*p* < 0.05	*p* < 0.001	*p* < 0.01	*p* < 0.001	*p* < 0.01	*p* < 0.05
	(d = 0.24)	(d = 0.52)	(d = 0.33)	(d = 0.66)	(d = 0.40)	(d = 0.27)
Grammatical score	*p* < 0.05	*p* < 0.001	*p* < 0.05	*p* < 0.001	*p* < 0.001	*p* < 0.05
	(d = 0.26)	(d = 0.49)	(d = 0.24)	(d = 0.72)	(d = 0.51)	(d = 0.28)
Pragmatic communication	*p* < 0.05	*p* < 0.001	*p* < 0.05	*p* < 0.001	*p* < 0.01	*p* < 0.05
	(d = 0.27)	(d = 0.56)	(d = 0.31)	(d = 0.68)	(d = 0.44)	(d = 0.29)

Note. n.s. = not significant. Effect-size benchmarks: According to Cohen, d ≈ 0.20–0.49 = small-to-medium effects; d ≈ 0.50–0.80 = medium-to-large effects.

**Table 9:** Multiple Regression Results.

Predictor	β (Standardized)	*p*	95% CI
Screen time	−0.29	<0.001	−0.36 to −0.21
Age	+0.47	<0.001	0.39 to 0.54
Educational content	+0.18	0.003	0.06 to 0.29
Parental mediation	+0.21	0.001	0.09 to 0.32

**Table 10:** Domain-Specific Multiple Regression Analyses Predicting Linguistic and Pragmatic Outcomes (*n* = 286).

Predictor	Receptive Vocabulary	Expressive Vocabulary	Semantic Fluency	Phonemic Fluency	Grammatical Score	Pragmatic Communication
Screen time	−0.24 **	−0.21 **	−0.27 ***	−0.22 **	−0.25 ***	−0.28 ***
Age	0.45 ***	0.43 ***	0.49 ***	0.46 ***	0.47 ***	0.48 ***
Educational content	0.16 *	0.14 *	0.18 **	0.15 *	0.17 **	0.19 **
Parental mediation	0.19 **	0.17 **	0.21 **	0.18 **	0.20 **	0.22 ***
R^2^	0.39	0.36	0.41	0.38	0.40	0.42
Adjusted R^2^	0.38	0.35	0.40	0.37	0.39	0.41

Note. Values are standardized β coefficients from multiple linear regression models. All predictors were mean-centered. * *p* < 0.05; ** *p* < 0.01; *** *p* < 0.001.

**Table 11:** Internal Consistency of Linguistic Measures (Cronbach’s α).

Variable	Cronbach’s α
Receptive vocabulary	0.78
Expressive vocabulary	0.84
Grammatical score	0.81
Pragmatic communication	0.89

## Data Availability

The data supporting the findings of this study are not publicly available due to privacy and ethical restrictions but can be provided by the corresponding author upon reasonable request.

## Supplementary Materials

The following supporting information can be downloaded at: https://www.mdpi.com/article/10.3390/children13010157/s1, File S1. Mean Profile of Standardized Composite Linguistic Performance by Age Group and Screen-Time Category; File S2. Differences in Linguistic and Pragmatic Variables as a Function of Screen-Time Category.

## Abbreviations

The following abbreviations are used in this manuscript:

PPVT	Peabody Picture Vocabulary Test
EVT	Expressive Vocabulary Test
COWAT	Controlled Oral Word Association Test

## References

[1] Canadian Paediatric Society, Digital Health Task Force (2019). Digital media: Promoting healthy screen use in school-aged children and adolescents. Paediatr. Child Health.

[2] Kar S.S., Dube R., Goud B.K.M., Gibrata Q.S., El-Balbissi A.A., Al Salim T.A., Fatayerji R.N.M.A.K. (2025). Impact of screen time on development of children. Children.

[3] Gath M., McNeill B., Gillon G. (2023). Preschoolers’ screen time and reduced opportunities for quality interaction: Associations with language development and parent–child closeness. Curr. Res. Behav. Sci..

[4] Tremolada M., Incardona R.M., Bonichini S., Taverna L. (2025). Use of technological devices in children aged 3–11 years: Possible effects on sleep and behavioral difficulties. Pediatr. Rep..

[5] Osser B., Toth C., Nistor-Cseppento C.D., Osser G., Miuța C.C., Ilia I., Iovanovici D.C., Aur C., Bondar L.I. (2025). Evaluating tech neck: A pilot study using a self-developed questionnaire on symptoms, posture, and preventive measures. Children.

[6] Madigan S., McArthur B.A., Anhorn C., Eirich R., Christakis D.A. (2020). Associations between screen use and child language skills: A systematic review and meta-analysis. JAMA Pediatr..

[7] Masek L.R., Edgar E.V., McMillan B.T.M., Todd J.T., Golinkoff R.M., Bahrick L.E., Hirsh-Pasek K. (2024). Building language learning: Relations between infant attention and social contingency in the first year of life. Infant Behav. Dev..

[8] Mallawaarachchi S., Burley J., Mavilidi M., Howard S.J., Straker L., Kervin L., Staton S., Hayes N., Machell A., Torjinski M. (2024). Early childhood screen use contexts and cognitive and psychosocial outcomes: A systematic review and meta-analysis. JAMA Pediatr..

[9] Muppalla S.K., Vuppalapati S., Reddy Pulliahgaru A., Sreenivasulu H. (2023). Effects of excessive screen time on child development: An updated review and strategies for management. Cureus.

[10] Marconi G.R., Osser B., Osser G., Miuța C.C., Toth C., Ardelean V.P., Dicu A., Toderescu C.D., Bondar L.I. (2025). Assessing nutritional knowledge and physical health among football players: A pilot study from three sports clubs in Western Romania. Sports.

[11] Patel R., McQueen E., Gold C. (2025). Balancing digital media exposure: Enhancing language and social development in early childhood. Pediatr. Rev..

[12] Panjeti-Madan V.N., Ranganathan P. (2023). Impact of screen time on children’s development: Cognitive, language, physical, and social and emotional domains. Multimodal Technol. Interact..

[13] Jing M., Ye T., Kirkorian H.L., Mares M.L. (2023). Screen media exposure and young children’s vocabulary learning and development: A meta-analysis. Child Dev..

[14] Bal M., Kara Aydemir A.G., Tepetaş Cengiz G.Ş., Altındağ A. (2024). Examining the relationship between language development, executive function, and screen time: A systematic review. PLoS ONE.

[15] Rayce S.B., Okholm G.T., Flensborg-Madsen T. (2024). Mobile device screen time is associated with poorer language development among toddlers: Results from a large-scale survey. BMC Public Health.

[16] Brushe M.E., Haag D.G., Melhuish E.C., Reilly S., Gregory T. (2024). Screen time and parent–child talk when children are aged 12 to 36 months. JAMA Pediatr..

[17] Takahashi I., Obara T., Ishikuro M., Murakami K., Ueno F., Noda A., Onuma T., Shinoda G., Nishimura T., Tsuchiya K.J. (2023). Screen time at age 1 year and communication and problem-solving developmental delay at 2 and 4 years. JAMA Pediatr..

[18] Osser B., Toth C., Osser G., Nistor-Cseppento C.D., Niculescu V., Bondar L.I. (2025). Student behavior and its association with multi-device addiction and back pain in Western Romania. Balneo PRM Res. J..

[19] Tan C.Y., Xu N., Liang M., Li L. (2025). Meta-analysis of associations between digital parenting and children’s digital wellbeing. Educ. Res. Rev..

[20] Modecki K.L., Goldberg R.E., Wisniewski P., Orben A. (2022). What is digital parenting? A systematic review of past measurement and blueprint for the future. Perspect. Psychol. Sci..

[21] Swider-Cios E., Vermeij A., Sitskoorn M.M. (2023). Young children and screen-based media: The impact on cognitive and socioemotional development and the importance of parental mediation. Cogn. Dev..

[22] Mathers S.J., Kolancali P., Jelley F., Singh D., Hodgkiss A., Booton S.A., Malmberg L.-E., Murphy V.A. (2025). Features of digital media which influence social interactions between adults and children aged 2–7 years during joint media engagement: A multi-level meta-analysis. Educ. Res. Rev..

[23] Slobodin O., Hetzroni O.E., Mandel M., Saad Nuttman S., Gawi Damashi Z., Machluf E., Davidovitch M. (2024). Infant screen media and child development: A prospective community study. Infancy.

[24] Bhutani P., Gupta M., Bajaj G., Deka R.C., Satapathy S.S., Ray S.K. (2024). Is the screen time duration affecting children’s language development? A scoping review. Clin. Epidemiol. Glob. Health.

[25] Bondar L.I., Osser B., Osser G., Mariș M.A., Piroș E.L., Almășan R., Popescu M.I. (2024). Ischemic heart disease as an important risk factor for depression—A case report. Appl. Sci..

[26] Namazi S.A., Sadeghi S. (2024). The immediate impacts of TV programs on preschoolers’ executive functions and attention: A systematic review. BMC Psychol..

[27] Bali C., Várkonyi G., Szabó M., Zsidó A.N. (2025). The impact of visual cues on reducing cognitive load in interactive storybooks for children. J. Exp. Child Psychol..

[28] Vedechkina M., Borgonovi F. (2021). A review of evidence on the role of digital technology in shaping attention and cognitive control in children. Front. Psychol..

[29] Taylor G., Sala G., Kolak J., Gerhardstein P., Lingwood J. (2024). Does adult–child co-use during digital media use improve children’s learning aged 0–6 years? A systematic review with meta-analysis. Educ. Res. Rev..

[30] Gowenlock A.E., Norbury C., Rodd J.M. (2024). Exposure to language in video and its impact on linguistic development in children aged 3–11: A scoping review. J. Cogn..

[31] Massaroni V., Delle Donne V., Marra C., Arcangeli V., Chieffo D.P.R. (2024). The relationship between language and technology: How screen time affects language development in early life—A systematic review. Brain Sci..

[32] Lakicevic N., Manojlovic M., Chichinina E., Drid P., Zinchenko Y. (2025). Screen time exposure and executive functions in preschool children. Sci. Rep..

[33] Maeneja R., Rato J., Ferreira I.S. (2025). How is the digital age shaping young minds? A rapid systematic review of executive functions in children and adolescents with exposure to ICT. Children.

[34] Naik V.S., Mathias E.G., Krishnan P., Jagannath V. (2025). Impact of social media on cognitive development of children and young adults: A systematic review. BMC Pediatr..

[35] Hinten A.E., Scarf D., Imuta K. (2025). Meta-analytic review of the short-term effects of media exposure on children’s attention and executive functions. Dev. Sci..

[36] Karani N.F., Sher J., Mophosho M. (2022). The influence of screen time on children’s language development: A scoping review. S. Afr. J. Commun. Disord..

[37] Gath M., Horwood L.J., Gillon G., McNeill B., Woodward L.J. (2025). Longitudinal associations between screen time and children’s language, early educational skills, and peer social functioning. Dev. Psychol..

[38] Sundqvist A., Majerle N., Heimann M., Koch F.-S. (2024). Home literacy environment, digital media and vocabulary development in preschool children. J. Early Child. Res..

[39] Liu S., Reynolds B.L., Thomas N., Soyoof A. (2024). The use of digital technologies to develop young children’s language and literacy skills: A systematic review. SAGE Open.

[40] Phillips B.M., Oliver F., Willis K.B. (2022). Engaging, explicit, and elaborated: An initial trial of media-enhanced preschool vocabulary instruction. Top. Lang. Disord..

[41] Cychosz M., Mahr T., Munson B., Newman R., Edwards J.R. (2023). Preschoolers rely on rich speech representations to process variable speech. Child Dev..

[42] Rhodes S.M., Stewart T.M., Kanevski M. (2020). Immediate impact of fantastical television content on children’s executive functions. Br. J. Dev. Psychol..

[43] Sundqvist A., Koch F.S., Birberg Thornberg U., Barr R., Heimann M. (2021). Growing up in a digital world—Digital media and the association with the child’s language development at two years of age. Front. Psychol..

[44] Pyne B., Asmara O., Morawska A. (2025). The impact of modifiable parenting factors on the screen use of children five years or younger: A systematic review. Clin. Child Fam. Psychol. Rev..

[45] Koch F.S., Barr R., Sundqvist A. (2025). The Joint Media Engagement Scale (JMES): An instrument for measuring shared media use with children aged 1 to 5 years old. Br. J. Dev. Psychol..

[46] Bayar M.E., Kulaksiz T., Toran M. (2025). How does parental media mediation regulate the association between digital parental awareness and the parent–child relationship?. Early Child. Educ. J..

[47] Xiao Y., Emmers D., Li S., Zhang H., Rule A., Rozelle S. (2025). Screen exposure and early childhood development in resource-limited regions: Findings from a population-based survey study. J. Med. Internet Res..

[48] Foulds K. (2023). Co-viewing mass media to support children and parents’ emotional ABCs: An evaluation of *Ahlan Simsim*. Early Child. Educ. J..

[49] Zhou X., Yu C.F., Zheng Z. (2025). Research on the relationship between parental media literacy and preschool children’s quality of learning in the new media environment. Front. Psychol..

[50] Levickis P., Eadie P., Mensah F., McKean C., Bavin E.L., Reilly S. (2023). Associations between responsive parental behaviours in infancy and toddlerhood, and language outcomes at age 7 years in a population-based sample. Int. J. Lang. Commun. Disord..

[51] Wengman J., Forssman L. (2025). Developmental relationships between early vocabulary acquisition, joint attention and parental supportive behaviors. Infancy.

[52] Karousou A., Economacou D., Makris N. (2023). Clustering and switching in semantic verbal fluency: Their development and relationship with word productivity in typically developing Greek-speaking children and adolescents. J. Intell..

[53] Hoyda J.C., Stewart H.J., Vannest J., Washington K.N., Moore D.R. (2025). Structural and pragmatic language skills in school-age children relate to resting state functional connectivity. Brain Imaging Behav..

[54] Brady N.C., Fleming K., Bredin-Oja S.L., Fielding-Gebhardt H., Warren S.F. (2020). Language development from early childhood to adolescence in youths with fragile X syndrome. J. Speech Lang. Hear. Res..

[55] Buteau-Poulin A., Gaudreau N., Desmarais C. (2025). Developmental language disorder at adolescence: Links between communication skills and self-efficacy ratings. Disabilities.

[56] Shokrkon A., Nicoladis E. (2022). The directionality of the relationship between executive functions and language skills: A literature review. Front. Psychol..

[57] Filipe M.G., Veloso A.S., Frota S. (2023). Executive functions and language skills in preschool children: The unique contribution of verbal working memory and cognitive flexibility. Brain Sci..

[58] Cadime I., Ribeiro I., Lorusso M.L. (2025). Cognitive and linguistic development in children and adolescents. Children.

[59] Bruce M., Savla J., Bell M.A. (2023). From terrible twos to sassy sixes: The development of vocabulary and executive functioning across early childhood. Dev. Sci..

[60] Khanani M.I., Khan M.R., Farooqi M.F., Fazal J., Aabideen Z., Alkuwaiti N.S. (2025). Digital media use and screen time exposure among youths: A lifestyle-based public health concern. Cureus.

[61] Liu J., Riesch S., Tien J., Lipman T., Pinto-Martin J., O’Sullivan A. (2022). Screen media overuse and associated physical, cognitive, and emotional/behavioral outcomes in children and adolescents: An integrative review. J. Pediatr. Health Care.

[62] Myers L.J., Arterberry M.E. (2022). Digital media and children under 3 years of age. Infant Behav. Dev..

[63] Kulkarni K., Waknis A.P. (2024). Screen-time and pragmatic development of toddlers and preschool children. J. Indian Speech Lang. Hear. Assoc..

[64] Mattie L.J., Fanta D. (2023). Joint engagement and early language abilities in young children with Down syndrome. Front. Psychol..

[65] Finders J., Wilson E., Duncan R. (2023). Early childhood education language environments: Considerations for research and practice. Front. Psychol..

[66] Capelli E., Grumi S., Vercellino L., Provenzi L. (2025). Joint attention and exogenous attention allocation during mother–infant interaction at 12 months associate with 24-month vocabulary composition. Front. Psychol..

[67] Sperber J.F., Hart E.R., Troller-Renfree S.V., Watts T.W., Noble K.G. (2023). The effect of the COVID-19 pandemic on infant development and maternal mental health in the first 2 years of life. Infancy.

[68] Ponti M. (2023). Screen time and preschool children: Promoting health and development in a digital world. Paediatr. Child Health.

[69] Barr R., Kirkorian H. (2023). Reexamining models of early learning in the digital age: Applications for learning in the wild. J. Appl. Res. Mem. Cogn..

[70] Sina E., Buck C., Ahrens W., Coumans J.M.J., Eiben G., Formisano A., Lissner L., Mazur A., Michels N., Molnar D. (2023). Digital media exposure and cognitive functioning in European children and adolescents of the I.Family study. Sci. Rep..

[71] Marciano L., Camerini A.L., Morese R. (2021). The developing brain in the digital era: A scoping review of structural and functional correlates of screen time in adolescence. Front. Psychol..

[72] Dong N., Zhou Y., Lei L., Lee T.M.C., Lam C.L.M. (2025). The longitudinal impact of screen media activities on brain function, architecture and mental health in early adolescence. Int. J. Clin. Health Psychol..

[73] Leonhardt C., Danielsen D., Andersen S. (2025). Associations between screen use, learning and concentration among children and young people in western countries: A scoping review. Child. Youth Serv. Rev..

[74] Feng X., Ren S., Shi P. (2025). The relationship and mechanism of screen time and academic performance among adolescents: An empirical study based on CEPS. Front. Public Health.

[75] Qu F., He S., Yu F., Gu C. (2025). The impact of electronic device use on learning quality in young children: The mediating role of executive function and the moderating role of parental mediation. Front. Public Health.

[76] Pedrotti B.G., Bandeira D.R., Frizzo G.B. (2024). Context of digital media use in early childhood: Factors associated with cognitive development up to 36 months of age. Infant Behav. Dev..

[77] Bonifacci P., Compiani D., Vassura C., Affranti A., Peri B., Ravaldini V., Tobia V. (2024). Home learning environment and screen time differentially mediate the relationship between socioeconomic status and preschoolers’ learning and behavioural profiles. Child Psychiatry Hum. Dev..

[78] Martinot P., Bernard J.-Y., Peyre H., De Agostini M., Forhan A., Charles M.A., Plancoulaine S., Heude B. (2021). Exposure to screens and children’s language development in the EDEN mother–child cohort. Sci. Rep..

[79] Liu H., Yi S., Li Y., Sun J., Xiao W., Bai Y., Chen Y. (2025). Association between screen exposure and language development delay during the COVID-19 pandemic for 18–72 months children in China: Evidence from a cross-sectional study. BMC Public Health.

[80] Dore R.A., Christakis D.A., Hale L. (2025). Digital Media Use and Language Development in Early Childhood. Handbook of Children and Screens.

[81] Chen Y., Cabrera N.J., Reich S.M. (2023). Mother–child and father–child “serve and return” interactions at 9 months: Associations with children’s language skills at 18 and 24 months. Infant Behav. Dev..

