# Supplementary material for: Screen Time, Digital Content Quality, and Parental Mediation as Predictors of Linguistic and Pragmatic Development: Implications for Pediatric and Preventive Health
Source: Children (Basel). 2026 Jan 22;13(1):157. doi: 10.3390/children13010157 (PMC12840120; doi:10.3390/children13010157)

Supplementary File S2. Differences in Linguistic and Pragmatic Variables as a Function of Screen-Time Category

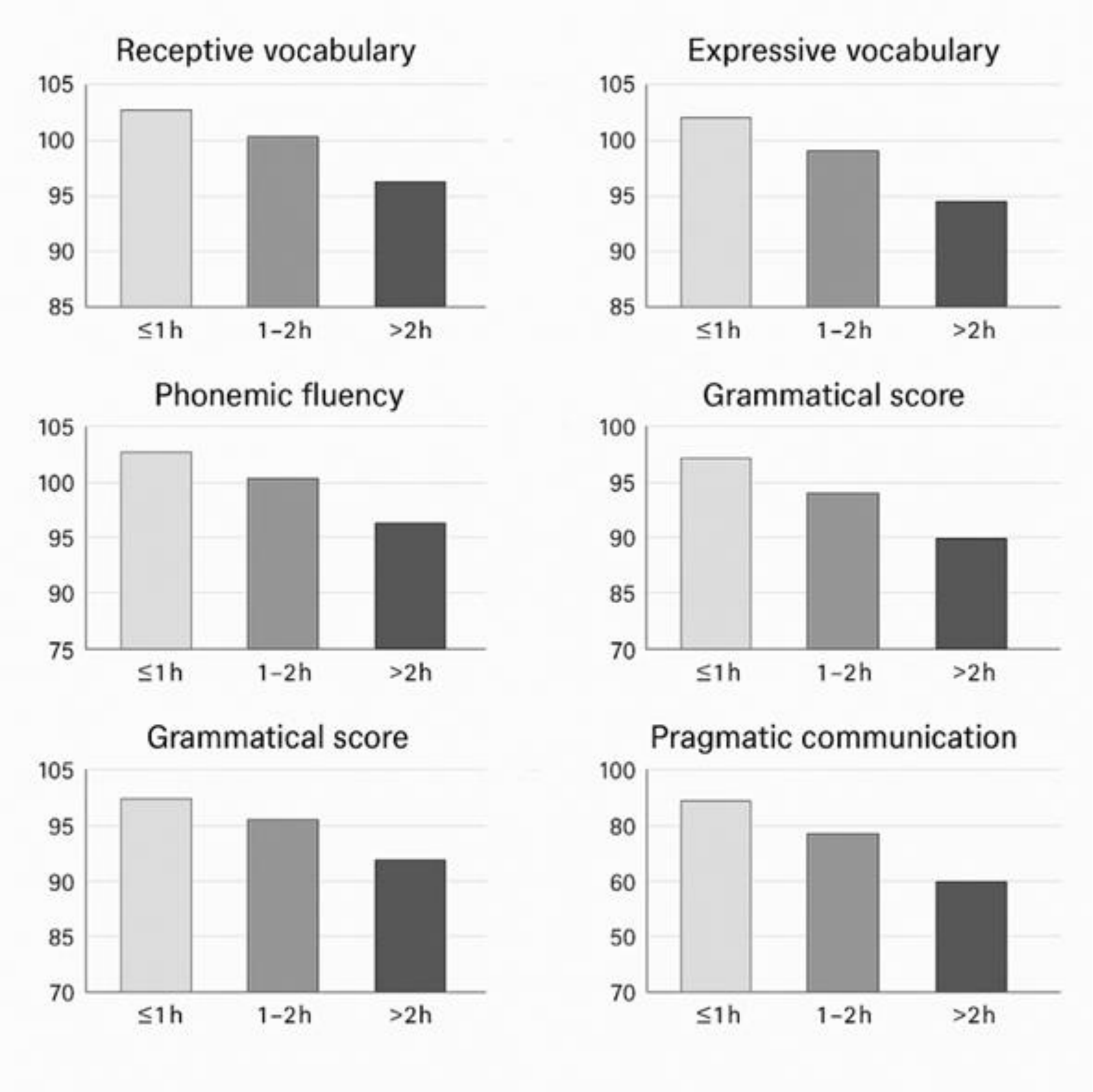

Supplement: Supplementary file 1 [file children-13-00157-s001.zip › File S2.pdf]
